# mTOR inhibition abrogates human mammary stem cells and early breast cancer progression markers

**DOI:** 10.1186/s13058-023-01727-z

**Published:** 2023-10-30

**Authors:** Hakim Bouamar, Larry Esteban Broome, Kate Ida Lathrop, Ismail Jatoi, Andrew Jacob Brenner, Alia Nazarullah, Karla Moncada Gorena, Michael Garcia, Yidong Chen, Virginia Kaklamani, Lu-Zhe Sun

**Affiliations:** 1 Department of Cell Systems and Anatomy, School of Medicine, The University of Texas Health Science Center at San Antonio, San Antonio, TX USA; 2 Department of Medicine, School of Medicine, The University of Texas Health Science Center at San Antonio, San Antonio, TX USA; 3 Department of Surgery, The University of Texas Health Science Center at San Antonio, San Antonio, TX USA; 4 Department of Pathology, School of Medicine, The University of Texas Health Science Center at San Antonio, San Antonio, TX USA; 5 Flow Cytometry Core Facility, The University of Texas Health Science Center at San Antonio, San Antonio, TX USA; 6 Department of Population Health Sciences, School of Medicine, The University of Texas Health Science Center at San Antonio, San Antonio, TX USA; 7 Greheey Children’s Cancer Research Institute, The University of Texas Health Science Center at San Antonio, San Antonio, TX USA

**Keywords:** Stem cells, mTOR, Rapamycin, Sirolimus, Progression markers, Mammary stem cells

## Abstract

**Background:**

Mammary physiology is distinguished in containing adult stem/progenitor cells that are actively amending the breast tissue throughout the reproductive lifespan of women. Despite their importance in both mammary gland development, physiological maintenance, and reproduction, the exact role of mammary stem/progenitor cells in mammary tumorigenesis has not been fully elucidated in humans or animal models. The implications of modulating adult stem/progenitor cells in women could lead to a better understanding of not only their function, but also toward possible breast cancer prevention led us to evaluate the efficacy of rapamycin in reducing mammary stem/progenitor cell activity and malignant progression markers.

**Methods:**

We analyzed a large number of human breast tissues for their basal and luminal cell composition with flow cytometry and their stem and progenitor cell function with sphere formation assay with respect to age and menopausal status in connection with a clinical study (NCT02642094) involving a low-dose (2 mg/day) and short-term (5–7 days) treatment of the mTOR inhibitor sirolimus. The expression of biomarkers in biopsies and surgical breast samples were measured with quantitative analysis of immunohistochemistry.

**Results:**

Sirolimus treatment significantly abrogated mammary stem cell activity, particularly in postmenopausal patients. It did not affect the frequency of luminal progenitors but decreased their self-renewal capacity. While sirolimus had no effect on basal cell population, it decreased luminal cell population, particularly in postmenopausal patients. It also significantly diminished prognostic biomarkers associated with breast cancer progression from ductal carcinoma in situ to invasive breast cancer including p16INK4A, COX-2, and Ki67, as well as markers of the senescence-associated secretary phenotype, thereby possibly functioning in preventing early breast cancer progression.

**Conclusion:**

Overall, these findings indicate a link from mTOR signaling to mammary stem and progenitor cell activity and cancer progression.

*Trial registration* This study involves a clinical trial registered under the ClinicalTrials.gov identifier NCT02642094 registered December 30, 2015.

**Supplementary Information:**

The online version contains supplementary material available at 10.1186/s13058-023-01727-z.

## Introduction

The ductal and lobular structures in the mammary gland are composed of epithelium consisting of myoepithelial cells forming the basal layer and luminal epithelial cells lining the lumen of ducts and lobules. The basal myoepithelial cells are found to highly express the cytokeratin genes *Krt5* and *Krt14*, while the inner luminal cell layer expresses *Krt8* and *Krt18*. The mammary epithelium undergoes cyclic expansion, differentiation, and regression during each menstrual cycle, which is driven by estrogen and progesterone hormonal signaling [1, 2].

In most organs, adult stem cells are responsible for the replenishment of cells to maintain tissue homeostasis. Mammary stem cells (MaSCs) and progenitor cells, however, control significant morphogenesis that occurs in the postnatal development and reproductive cycles in the mammary gland epithelium. Multipotent MaSCs in mice were first demonstrated to exist through transplantation studies of tissue fragments and then later as single cells, which were able to regenerate a whole mammary gland [3]. These MaSCs, also called mammary repopulating units (MRUs), were found to be co-isolated with the basal myoepithelial population of cells through flow cytometry sorting of hematopoietic lineage-negative (Lin^−^) CD24^+^ CD49f^high^ CD29^high^ cells. On the other hand, luminal epithelial cells that are Lin^−^CD24^high^ CD49f^+^ CD29^+^ contain luminal progenitors, which are proliferative but show no regenerative potential [4–6]. Such studies provided evidence of a multipotent population of MaSCs in mice, which were later confirmed to exist in human breast tissue by transplantation studies in humanized mouse mammary fat pads [7].

The human mammary gland epithelial populations can be sorted into three distinct groups: basal myoepithelial (BM) cells expressing Lin^−^ EpCAM^low/−^ CD49f^high^, luminal epithelial progenitors (LP) expressing Lin^−^ EpCAM^high^ CD49f^low^, and mature luminal (ML) cells expressing Lin^−^EpCAM^high^ CD49f^low/−^ [5]. Studies with primary and cultured human mammary epithelial cells from women of different ages showed a significant decrease of the fraction of myoepithelial population in older women, while the fraction of luminal epithelial populations was comparatively increased. A molecular expression shift was also observed in subjects over 55 years of age by an increase in CD49f and keratin 14 expression in the luminal cells, indicating a luminal to basal trans-differentiation during aging [8]. However, the effect of age on the frequency of human MaSCs and luminal progenitors is not well known.

Strong correlations between stem/progenitor cell activity and the onset of cancer have been reported [9]. Breast cancer itself is an assembly of various cancer subtypes with distinct molecular, physiological, and clinical characteristics. This heterogeneity of tumor types is likely a reflection of the cells that originate the transformation into a cancer-like state, which in some cases implicates MaSCs and progenitor cells. Ductal carcinoma in situ (DCIS) is the most commonly diagnosed breast neoplastic lesion, comprising 20% of all neoplastic lesions detected with mammography screening [10]. It is thought to be a non-obligate precursor of invasive carcinoma and likely derives from a single cell origin. DCIS has been shown to contain stem-like cell populations and implicates a possible relationship between early carcinogenesis and MaSCs [11]. Among the DCIS lesions found in women, those that show high expression of p16^Ink4A^ (p16), COX-2, and Ki67 are at a higher risk of developing subsequent invasive cancer [12, 13]. This expression set of p16^+^COX-2^+^Ki67^+^ has been suggested to be an effective biomarker of cancer progression and is linked to a myoepithelial basal cell origin in high-grade DCIS [14]. The modulation of the early cancer progenitors could be a valuable tool in breast cancer prevention, and active adult stem cells would be a likely entry point for such treatment.

mTOR signaling pathways, which include Akt and PI3K, are among the most commonly altered pathways leading to tumorigenesis in breast cancer [15]. This leads to an overexpression and/or aberrant phosphorylation of downstream targets such as p-70S6K and p-4E-BP1, which have been implicated in driving carcinogenesis [16]. Regulating the activity of mTOR signaling could provide a means for preventing the age-associated onset of transformation in epithelial cells. Thus, mTOR inhibitors such as rapamycin and its analogs may be promising chemo-preventive agents. Rapamycin has proven to be a reliable agent in increasing lifespan in nearly all mammalian model organisms studied [17]. Part of this ability may reside in the role that mTOR signaling has in promoting cellular senescence, which is increased during aging [18]. Senescence is thought to be a mechanism to prevent cancer by stalling the cell cycle, yet a major marker of senescent cells, p16, has been implicated in advancing tumor progression. As a tumor suppressor, p16 has generally been observed to be silenced in cancers yet paradoxically has also been observed to be highly expressed in progressive tumors [12, 13, 19]. Another role by which senescent cells promote age-related disorders is through the senescence-associated secretory phenotype (SASP), which promotes an inflammatory environment implicated in promoting mammary epithelial tumorigenicity [20]. mTOR signaling itself is a regulator of SASP by promoting the phosphorylation of the protein 4E-BP1, which enhances the translation of SASP components [21]. Among the various secreted factors that are thought to be able to induce senescence is the tumor necrosis factor (TNF) alpha produced by CD4 + T helper 1 cells in pancreatic and breast cancer [22]. Another common SASP factor is the cytokine interleukin 6 (IL-6) that is induced during senescence as a response to replicative stress, oncogene activation, or DNA damage [23]. The mechanism by which mTOR inhibition increases lifespan is still to be determined, but the delay and/or prevention of age-related disease would account for this effect. In cancer prone mice, mTOR inhibition by rapamycin was able to increase the lifespan, which is partially accomplished by the delay of tumor development in multiple tissue types through the treatment [24].

In this study, age-associated effects on mammary epithelial populations and their associative stem/progenitor populations were defined through two cohorts of human breast tissue samples. One was a collection of tissue samples from Cooperative Human Tissue Network (CHTN) and the other one was from a clinical trial (NCT02642094) with rapamycin/sirolimus treatment in patients with DCIS, atypical ductal hyperplasia (ADH), or lobular carcinoma in situ (LCIS) in our university. Our study revealed changes in certain epithelial populations and MaSC frequency according to age and menopausal status. This has relevance as age is the greatest correlative in breast cancer development other than sex and menopause predominantly occurs during the later period of the woman’s life [25]. Additionally, female reproductive hormone signaling through estrogen and progesterone is thought to be major factors in cancer initiation as they both are involved in nuclear and extracellular signaling that is thought to drive the majority of breast cancer types [26].

We also show that mTOR inhibition alters certain mammary epithelial populations, MaSC frequency, and passage potential of luminal-derived spheres. Disease-associated progression markers and SASP markers were also significantly reduced by mTOR inhibition. Together, these data for the first time show a novel approach for modulating the activity of MaSCs and luminal progenitors and inhibiting progression in early-stage human breast cancer.

## Patients and methods

### Patient eligibility

We acquired primary tissue samples from patients diagnosed with noninvasive lesions as detected by clinical pathology at the University of Texas Health Science Center at San Antonio (UTHSCSA) (San Antonio, TX). Inclusion criteria of this study carried out at the Mays Cancer Center at UT Health San Antonio included women of at least 18 years of age with confirmed menopausal status who were diagnosed with DCIS, LCIS, ADH, atypical lobular hyperplasia (ALH) lesions detected in biopsy by pathology and scheduled for mastectomy or lumpectomy. Patients were required to have normal organ and bone marrow function, on contraception if of child-bearing status, and were not pregnant throughout the treatment period.

Exclusion criteria included: concomitant treatment for their DCIS, LCIS, ALH or ADH diagnosis, active infection requiring therapy, immunocompromised health, or allergies to rapamycin and its analogs.

The fresh adjacent non-tumor breast tissues for both control and sirolimus-treated patients were collected through the local University Health system. Adjacent non-tumor mammary tissues from patients with breast cancer were also collected from the Cooperative Human Tissue Network (CHTN). They were minced in RPMI medium, digested, and prepared for single-cell sorting according to previously established methods [27].

## Treatment

**Fig. 1:**
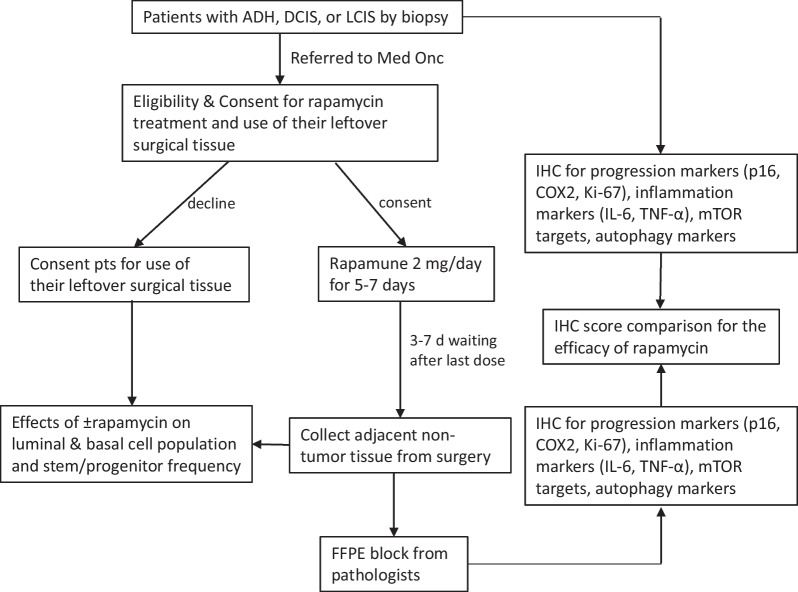
Flow chart of clinical trial study design methods

The primary research objectives of this non-random, open-label, phase II, window of opportunity trial (NCT02642094) were to investigate a possible reduction of MaSCs and/or malignant markers in DCIS, LCIS, or ADH in patients receiving an oral rapamycin (sirolimus) for 5–7 days at 2 mg/day. Pathological and molecular biomarkers associated with breast cancer aggressiveness were assessed by a pathologist, and the features of MaSCs were determined by the research laboratory for determining the effect of sirolimus. All experiments were performed without full blinding. The above-mentioned analyses (Fig. 1) were performed for each patient’s tissue if there was enough tissue sample.

## Antibodies

Antibodies used for cell sorting comprised of biotin-labeled anti-CD31 (Catalog #13–0319-82), anti-CD45 (#13–0459-82), anti-CD235a (#13–9987-82, Invitrogen, Carlsbad, CA) and biotin labeling with anti-biotin brilliant violet (BV) 605 streptavidin (# 405,229, Biolegend, San Diego, CA). Fluorescein isothiocyanate (FITC) labeled anti-EpCAM (#60136FI, STEMCELL Technologies Inc., Vancouver, Canada), while phycoerythrin (PE) labeled anti-CD49f antibodies (#313,612, Biolegend). IHC antibodies include the following: phospho 4E-BP1 (Ser65/Thr70) (#PA5-104,563, Invitrogen), phospho 4E-BP1(Thr37/46) (#2855S, Cell Signaling, Danvers, MA), phospho p70S6K1 (Thr389/412) (#PA5-104,842, Invitrogen), COX2 (#15,191, Abcam, Cambridge, MA.), p62 (#91,526, Abcam), Ki67 (#790–4286, Ventana, Tucson, AZ), p16 (#PA1-30,670, Invitrogen), LC3B (#NB100-2220, Novus Biologicals, Littleton, CO), IL-6 (#MAB2061, R&D Systems), and TNFα (#NBP1-19,532, Novus Biologicals).

## Cell labeling and flow cytometry

Labeling was proceeded by incubating the cells suspended in a solution of phosphate-buffered saline (PBS) supplemented with 2% fetal bovine serum on ice for 15 min with the biotinylated CD31, CD45, and CD235a antibodies mixture (~ 3 µg/mL each) and then washed with the PBS solution. Labeling was then finalized by incubation with anti-EpCAM labeled with FITC, anti-CD49f labeled with PE, and streptavidin-BV605 on ice for 15 min and washed with the PBS solution again. Cells were sorted according to the gates illustrated in (Additional file 1: Fig. S1) using a FACS Aria-IIIu (BD Biosciences) in which the basal cells (CD49f^high^ EpCAM^low/−^), luminal progenitors (CD49f^low^ EpCAM^high^), and mature luminal populations (CD49f^low/−^ EpCAM^high^) were isolated from Lin^−^ cells.

## Sphere formation assay (SFE) and serial passaging

Following 6 days after plating sorted cells, the individual sphere numbers with diameter greater than 25 μm were counted under a phase contrast microscope. SFE per thousand value was quantified by the following formula: $SFE=\frac{\#spheres}{plated\; cells} X 1000$

Serial passaging was performed by transferring the spheres into BD Matrigel (BD Biosciences 356,234) to form organoids in complete EpiCult™-B Human Medium for 3D-Organoid culturing for 7 days [27]. Then, all the organoids were dissociated with trypsin and plated in a 96-well plate at 5,000 cells per well (controls n = 11, treatment n = 5) at 2 wells/sample in Matrigel. Between each passage, the number of organoids was counted and the mean was calculated. After each passage, the organoids were re-dissociated and re-plated at 5,000 cells per well.

## Immunohistochemistry

Patient tissue was fixed for 24 h in 10% neutral-buffered formalin, dehydrated in ethanol, and embedded in paraffin wax. Tissue sections were cut to 5 μm on glass slides, de-paraffined, and rehydrated by graded ethanol solutions. Antigen retrieval was executed by heating in sodium citrate (10 mM; pH 6.0; 95 °C) for 10 min and allowed to return to room temperature for another 10 min. Endogenous peroxidase reaction was prevented by incubating sections with 3% H_2_O_2_ for 15 min, while nonspecific binding was blocked with 10% goat serum for 30 min at room temperature. The sections were incubated with primary antibodies overnight with phosphate-buffered saline and 0.025% Triton solution (PBST) and 5% goat serum in a humidified chamber at 4 °C. Samples were then washed twice with PBST. Biotin-conjugated secondary antibodies were incubated for 1 h at room temperature. After washing, the samples were incubated with streptavidin–horseradish peroxidase for 30 min and counterstained with hematoxylin for 2 min before dehydration and mounting. Slides were imaged using the brightfield microscope Aperio VERSA and then analyzed using Aperio ImageScope software (Leica Biosystems Inc., Buffalo Grove, IL, USA) for staining quantification using customized nuclear v9 or cytoplasmic v2 algorithm to measure the positive pixels of staining [28]. A negative control slide, which was put through the whole staining process except the primary antibody staining step, was used to set the baseline as no staining. Three representative high-power fields in each stained slide were selected for staining intensity scoring with four scales by a selected algorithm: 0 = no staining, 1 +  = weak staining, 2 +  = moderate-to-strong staining, and 3 +  = cells stained with strong intensity. A single intensity score-weighted value was computed for each high-power field by summation of the products of each intensity score multiplied by the percent of the scored area.

## Statistics

Unpaired t-tests were used for epithelial population statistical analysis and statistical significance for SFE between control and treatment groups. Two-way ANOVA was used for statistical significance for organoid serial passaging. Statistical comparison of the average IHC staining value between pre- and post-treatment samples was performed with 2-way repeated-measure ANOVA for phospho-p70, phospho-4E-BP1, COX-2, p16, and p21 and with paired t-tests for Ki67, p62, and LC3.

Availability of case sample size for the clinical trial was determined by taking a 60% enrollment rate of DCIS cases at the Mays Cancer Center from 2008 to 2013, which was roughly 60 patients. We used Mantel–Haenszel test for categorical data of immunohistochemical staining intensity scores ranging from 0 to 4 to determine sample size. To reach statistical power > 80%, a patient group size of 31 or more was required for us to detect the difference of a score of 0.3 between untreated biopsy and treated surgical specimens with the significance level targeted at 0.05 for one-sided Mantel–Haenszel test (PASS 14, NCSS, LLC, Kaysville, UT).

Multivariate analysis of variance was performed through an ANOVA statistical analysis to detect significant factors in a multi-factor model (R, ANOVA function). In the model, six (6) factors (i.e., treatment, menopause status, race, ethnicity, diagnosis, and ER/PR status) and their interactions were considered to examine five response variables individually (Basal myoepithelial (BM) cells, luminal progenitor (LP) cells, mature luminal (ML) cells, sphere formation efficiency (SFE) of basal (Basal SFE) cells, or of luminal (Lum SFE) cells).

## Results

### Patient characteristics

A total of 27 patients agreed to undergo treatment and successfully completed the regiment. The control group included 12 patients with DCIS or ADH, who declined to participate in the sirolimus study but agreed to donate their tissues, and 6 patients with invasive ductal carcinoma for a total of 18 controls (Additional file 6: Table S1). The average age of the controls was 52 years old with an age range of 33–76 years, while the treatment groups was 59 years old (range 42–79 yrs) (Table 1).

**Table 1:** Summary of patient characteristics

Treatment group	Number of patients	Mean age (range)	Pre- vs post menopausal ratio	%White	% Other race	% Hispanic White	% ER +	% PR +	% Undetermined hormonal status
Control	18	53(33–76)	8:10	88	12	60	67	50	22
Sirolimus	27	59(42–79)	6:21	85	15	62	89	81	11
P-value		0.0681	0.1883	> 0.9999		0.5338	0.1294	0.0772	0.4002

The majority of patients were white (88% controls, 85% treatments) with many patients identified as Hispanic (60%, 62%). Other racial identities comprised 12% of the controls and 15% of the treated patients. Overall, the higher percentage of Hispanic women is indicative of the demography in south-central Texas area where 65% is Hispanic [29]. Ten of the control patients had undergone menopause, while twenty-one of the treated patients were postmenopausal. Estrogen receptor (ER) and progesterone receptor (PR) status was identified as 67% (ER +) and 50% (PR +) with 22% unidentified in excised pre-cancer tissue of the control group, whereas the treated patient’s tissues were 89% (ER +) and 81% (PR +) with 11% unidentified. All parameters were found to not be significantly associated between patient groups based on unpaired t-test for mean ages and Fisher’s exact test for all other comparisons.

## Toxicity

Overall, the treatment was well tolerated by the patients with no adverse effects (AEs) at or above grade 3 (Table 2).

**Table 2:** Adverse effects of sirolimus treatment

Adverse effects (AEs)	All	% Patient with AEs	Grade 3–4
Diarrhea	5	20.8	0
Fatigue	3	12.5	0
Headache	9	37.5	0
Nausea	8	33.3	0
Pruritus	2	8.3	0
Rash	2	8.3	0
Stomach pain	3	12.5	0

Lower-grade toxicities included headaches (37.5% of patients), nausea (33.3%), and diarrhea (20.8%) representing the top three AEs.

## Effect of sirolimus treatment on human mammary epithelial cell populations

**Fig. 2:**
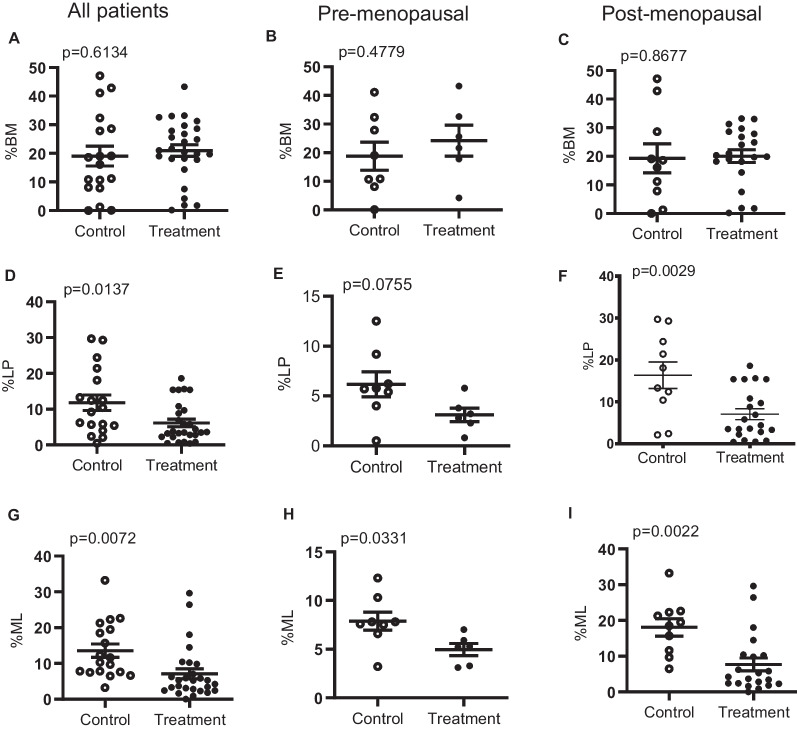
Effect of sirolimus treatment on mammary epithelial populations. Scatter plots for comparison of BM populations between sirolimus-treated patients (*n* = 27) and controls (*n* = 18) **A**, between treatment (*n* = 6) and control (*n* = 8) groups of pre-menopausal patients **B**, and between treatment (*n* = 21) and control (10) groups of postmenopausal patients **C** using unpaired t-tests. LP populations are shown between groups **D** and in pre- and postmenopausal derived patients **E & F**. ML populations are shown between groups **G** and in pre- and postmenopausal derived patients **H & I**

In order to investigate whether mTOR inhibition would have an effect on the epithelial populations, we utilized the epithelial membrane marker proteins, CD49f and EpCAM, for flow cytometry analyses in cells derived from tissues of sirolimus-treated patients and controls. We observed no statistically significant changes in BM cell population between controls and treated patients (Fig. 2 A).

No differences were observed either when separated by their menopausal status (Fig. 2 B & C). However, the LP cell population was significantly decreased (*P* < 0.05) in the treatment group when compared to the control group (Fig. 2 D). When separating the patients by menopausal status, we found that the difference was not primarily within the pre-menopausal group (*P* > 0.05) but instead mainly derived from the postmenopausal patients (*P* < 0.05) (Fig. 2 E & F). ML cells also showed a response to mTOR inhibition with a decrease in population (*P* < 0.05) (Fig. 2 G). The inhibitory effect was observed in both menopausal groups (*P* < 0.05) (Fig. 2 H & I). To better understand the possible effects of age and menopause on mammary epithelial cells, primary non-tumor breast tissue samples were collected from two sources: 39 samples from CHTN (24–79 years old) and the 18 samples from the control arm of the sirolimus trial. No significant differences were observed in the percentages of basal, luminal progenitor, and mature luminal epithelial populations with increasing age or menopausal status (Additional file 2: Fig. S2).

Thus, the short-term sirolimus treatment can reduce luminal cell populations, both mature and progenitor in the postmenopausal women, with no effect on BM cell population, and that this effect is not linked to differences in patient age or hormonal status.

## Sirolimus treatment abrogated MaSC activity and self-renewal capacity of luminal progenitors

**Fig. 3:**
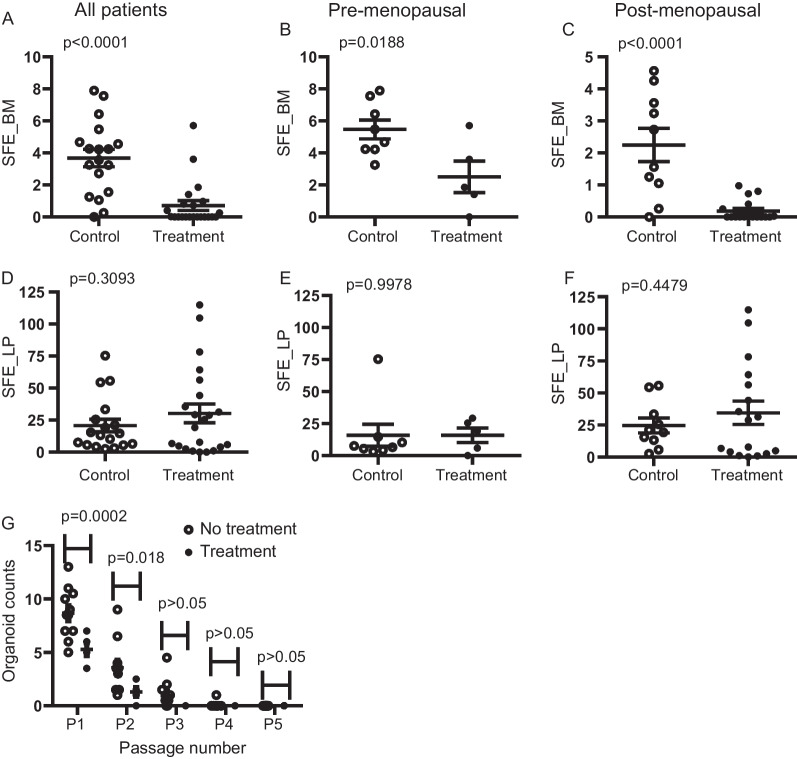
Effect of sirolimus treatment on SFE of BM and LP cells**.** **A** Scatter plots of the SFE of FACS-sorted BM cells of control (*n* = 18) and treated (*n* = 22) patient-derived tissue for all groups with unpaired t-test. Pre-menopausal **B** and postmenopausal patients **C** are compared between treatment (*n* = 5, *n* = 17) and control groups (n = 8, n = 10). **D** Scatter plots of sorted LP cells are also shown for control (*n* = 18) and treated (n = 22) patient groups including pre-menopausal **E** and postmenopausal patients **F** comparisons between treatment (*n* = 5, *n* = 17) and control groups (n = 8, *n* = 10). **G** Serial passaging of LP cells in 3D organoid culture for comparison of self-renewal capacity between control (*n* = 11) and treatment groups (*n *= 5) for a duration of 5 passages and differences were evaluated by 2-way ANOVA

We have previously shown that MaSCs and luminal progenitor cells can form mammospheres in suspension culture, which undergo further proliferation and differentiation to form solid or hollow 3D organoids in Matrigel [30]. Thus, this sphere formation assay can quantitatively measure the frequency and self-renewal capacity of MaSC and luminal progenitor cells and was used to investigate the effect of mTOR inhibition on the activity of MaSCs and luminal progenitors. Figure 3A shows a clear reduction of sphere-forming MaSCs in the BM cell population (*P* < 0.0001) indicating abrogation of stem cell activity by sirolimus treatment.

Notably, while SFE of the BM cells from the pre-menopausal patients showed a modest reduction (*P* < 0.05) by sirolimus treatment (Fig. 3B), the BM cells from most postmenopausal patients had no sphere-forming MaSCs after sirolimus treatment (Fig. 3C), indicating that the postmenopausal MaSCs appear more sensitive to mTOR signaling disruption than MaSCs from pre-menopausal patients. Interestingly, the sphere formation capacity of the BM cells was also significantly reduced during aging in the CHTN cohort and after menopause in the UT control cohort (Additional file 3: Fig. S3 A-C).

In contrast to BM cells, the SFE of LP cells was not significantly altered during aging and after menopause (Additional file 3: Fig. S3 D-F). Sirolimus treatment also showed no significant effect on the SFE of LP cells (Fig. 3D), regardless of the menopausal status (Fig. 3 E & F), suggesting that sirolimus treatment did not change the activity and/or the number of luminal progenitors. Since mature luminal cells do not yield spheres in a low attachment environment, there were no SFE data available to observe [27]. To determine whether self-renewal capacity of luminal progenitors was altered by sirolimus treatment, a given number of cells dissociated from the primary 3D organoids formed by luminal progenitors of postmenopausal patients in Matrigel were serially passaged in 3D organoid culture. Interestingly, the organoid formation efficiency by the luminal progenitor-derived cells from patients in the control group was significantly higher than that by the luminal progenitor-derived cells from patients in the treatment group in the first two passages with all sirolimus-exposed cells failing to form spheres by the third passage, while seven of the eleven controls continued to form organoids (Fig. 3G). Therefore, sirolimus treatment significantly abrogated the number and/or proliferative activity of both MaSCs and luminal progenitors, albeit with more potent inhibition on MaSCs, particularly in postmenopausal women. Multivariate analysis of variance also showed that rapamycin treatment significantly reduced percent LP and ML cells and SFE of BM cells after controlling the other 5 factors: menopausal status, race, ethnicity, diagnosis, and ER/PR status (Additional file 7:  Table S2). Menopause and ER/PR status are also independent factors contributing to significant changes of percent LP and SFE of BM cells. In addition, we also observed significance test effect on SFE of BM cells due to interactions between “Treatment and ethnicity” (*P* = 0.0075), “Menopausal status and ER/PR” (*P* = 0.044), and “Treatment and ER/PR” (*P* = 0.0061). However, a close examination indicated that the significant interactions were due to low sample size in each sub-category. Thus, they do not affect our overall conclusion about the inhibitory effect of rapamycin treatment on the SFE of BM cells.

## Sirolimus treatment reduced phosphorylation of mTOR kinase targets

**Fig. 4:**
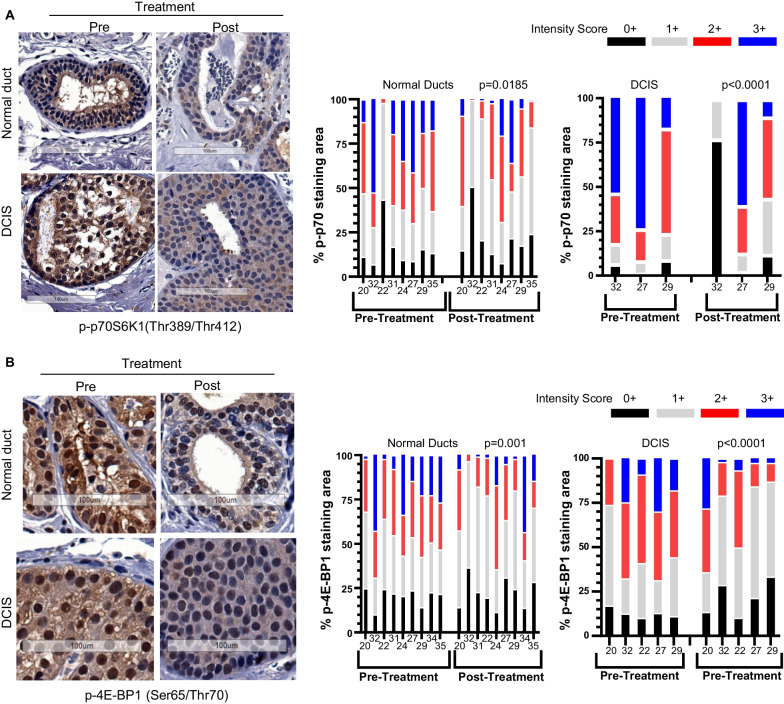
Sirolimus treatment reduced p70S6K1 and 4E-BP1 phosphorylation **A** Representative mammary tissue section images (left) from paired pre- and post-treatment samples by patient number show immunohistochemically stained phosphorylated p70S6K1 (Thr389/Thr412) in normal ducts and DCIS lesions; nuclei are stained blue. The stained protein expression between paired tissue samples of pre- and post-treatment in normal (middle) (n = 8) and DCIS tissue (right) (n = 3) was scored in three representative high-power fields per tissue section as 0, no staining; 1 + , weak diffuse cytoplasmic staining, 2 + , moderate-to-strong granular cytoplasmic staining, and 3 + , cells stained with strong intensity. The Y-axis shows the % of each scored area. **B** Representative images of stained phosphorylated 4E-BP1 (Ser65/Thr70) and quantitative analysis of its expression in pre- and post-treatment normal (n = 9) and DCIS tissue (n = 5). An intensity score-weighted single value was calculated for each high-power field and used for comparison between pre- and post-treatment by 2-way ANOVA. Scale bar, 100 µm. Note: the fewer DCIS paired cases than normal duct paired cases were due to the absence of DCIS lesions in some tissue sections

To ascertain the effectiveness of mTOR inhibition by sirolimus in the patient tissues, phosphorylation of known targets of mTOR complex 1 (mTORC1) kinase was assessed by standard immunohistochemistry (IHC). The phosphorylation sites of p70S6K1 (Thr389/Thr412) and 4E-BP1 (Ser65/Thr70) have been described as being strongly inhibited by rapamycin treatment [31]. Figure 4A shows a reduction of stained phosphorylated p70S6K1(Thr389/Thr412) (p-p70S6K1) by sirolimus treatment when we compared the staining intensity between pre- and post-treatment breast tissues in both normal ducts and DCIS lesions of the same patient.

Quantitative analysis of staining intensity and area (see Materials and Methods for details) also showed that the reduction of p-p70S6K1 in both normal ducts and DCIS lesions was statistically significant (*P *< 0.05) (Fig. 4A). Likewise, the phosphorylation of 4E-BP1 (Ser65/Thr70) showed a strong response to sirolimus treatment in normal and DCIS tissue (Fig. 4B). We did not observe an increase in autophagy, which is thought to be induced by mTOR inhibition, as the markers of autophagosome, p62 and LC3B foci, did not increase when paired pre- and post-treatment tissues were compared (Additional file 4; Fig. S4A & B).

Instead, LC3B foci in normal ductal cells were significantly decreased (*P* < 0.05) after sirolimus treatment.

## Sirolimus treatment reduced early-stage breast cancer progression markers

We next measured the expression of p16, COX2, and Ki67 in patient-derived tissues before and after sirolimus treatment to investigate the potential of the treatment for the prevention of invasive breast cancer. Of the three, p16 is perhaps the strongest prognostic marker for early-stage breast cancer progression [14], in part because high cytosolic p16 level is believed to be tumor-promoting and high nuclear p16 level causes cellular senescence resulting in SASP, which can

**Fig. 5:**
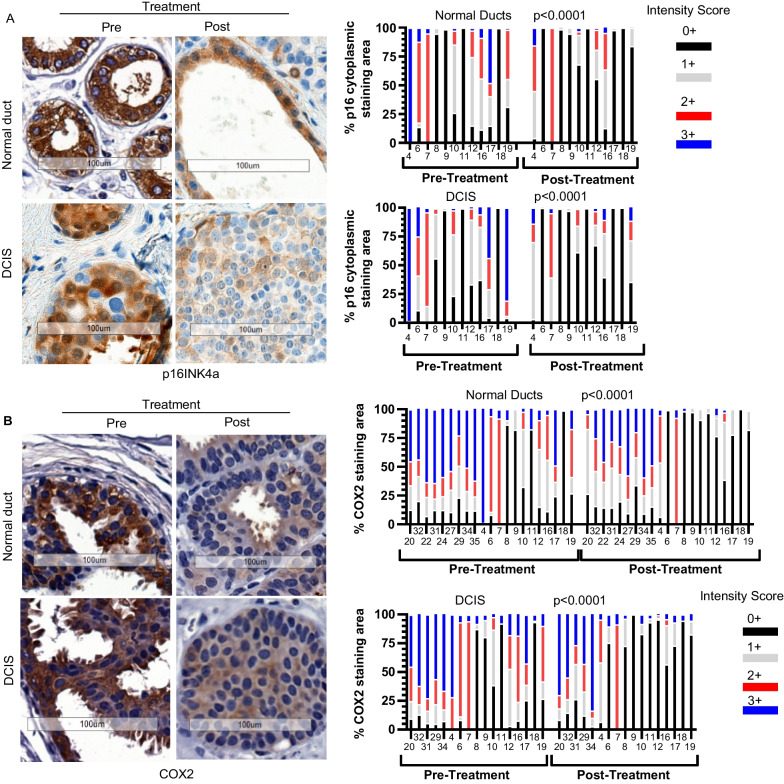
Sirolimus treatment reduced cancer progression markers (**A**) Representative mammary tissue section images **(left)** from paired pre- and post-treatment samples show expression of cytoplasmic & nuclear p16 protein in normal ducts (n = 12) and DCIS lesions (n = 12). Scale bar, 100 µm. Cytoplasmic p16 protein expression was quantified in pre- and post-sirolimus treated patients in normal (upper right) and DCIS (bottom right) tissue. (**B**) COX2 protein staining by IHC and quantitative expression analysis in normal (n = 21) and DCIS tissues (n = 17) from pre- and post-treatment samples. Statistical analysis was performed by 2-way ANOVA. Scale bar, 100 µm

also promote tumor progression [32, 33]. Significantly, we found that both cytoplasmic and nuclear p16 expression were remarkably reduced after treatment in both adjacent normal and DCIS tissue (Fig. 5A and Additional file 5: Fig. S5).

Staining quantification also showed a statistical difference (*P* < 0.0001) between paired samples of pre- and post-treatment tissues. COX2 staining showed a similar response (*P* < 0.0001) to that of p16 and was reduced in both normal and DCIS tissue types (Fig. 5B).

## Effect of sirolimus treatment on proliferation and cell cycle markers

**Fig. 6:**
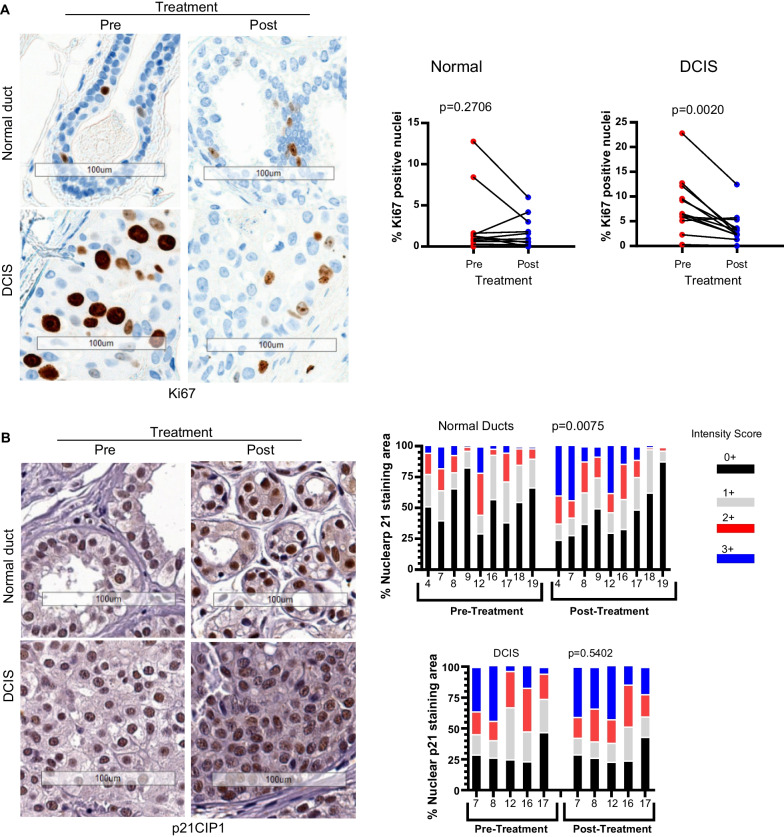
Effect of sirolimus treatment on proliferation and cell cycle markers **A** Ki67 staining by IHC and quantitative analysis of positively stained nuclei of pre- and post-sirolimus treatment in normal (n=12) and DCIS tissue (n=12). **B** p21 nuclear staining by IHC and quantitative expression analysis in normal (n=9) and DCIS tissues (n=5) from pre- and post-treatment samples. Significance was evaluated by paired t-test for Ki67 and 2-way ANOVA for p21. Scale bar, 100 μm.

Percent of nuclear positive Ki67 staining, a proliferation marker, was found to be significantly (*P* < 0.01) reduced in DCIS tissue after sirolimus treatment, whereas there was no significant change in adjacent normal tissue due to very few normal cells with Ki67 staining (Fig. 6A).

Since the effects of sirolimus were observed to inhibit proliferation, we then investigated the expression of the cell cycle regulator p21^Cip1^ (p21) for changes, which might indicate an alteration in cell cycle progression. Quantitative analysis of nuclear p21 level revealed a significant increase of p21 expression in adjacent normal epithelial cells after sirolimus treatment with no significant change in DCIS cells (Fig. 6B). Thus, mechanisms other than the two cell cycle inhibitors, p16 and p21, appear to mediate the inhibition of cell proliferation by sirolimus in DCIS lesions.

## SASP factors were decreased by sirolimus treatment in normal and DCIS ducts

**Fig. 7:**
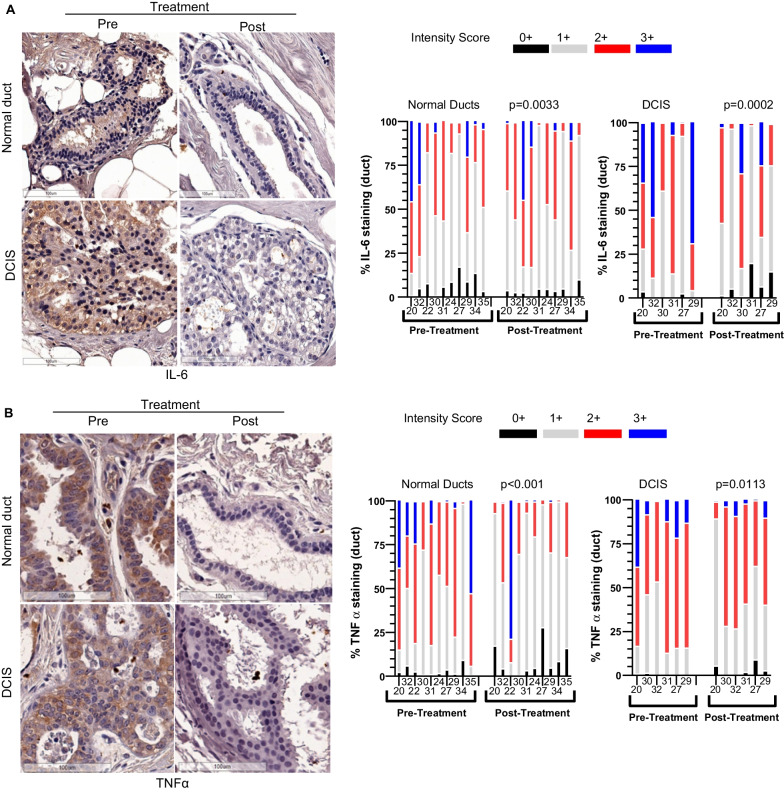
Sirolimus treatment reduced SASP factors **A** IL-6 and **B** TNFα staining by IHC and the quantitative expression analysis of their positive staining in pre- and post-sirolimus-treated normal (n=12) and DCIS tissues (n=6). Significance was evaluated by 2-way ANOVA. Scale bar, 100 μm.

Given that p16 and COX2 play a key role in cellular senescence and inflammation respectively, their significant reduction after sirolimus treatment appears to indicate an attenuated SASP and inflammatory response, which should contribute to the anti-malignant activity of sirolimus. We further validated this possibility by measuring the expression of two proteins consistently associated with SASP [23]. IL-6 and TNFα levels were measured in both normal and DCIS ducts through IHC. Both were significantly reduced (P < 0.05) in both normal ducts and DCIS lesions in post-treatment tissue samples compared to the paired pre-treated ones (Fig. 7A and B).

## Discussion

Age-related changes in normal human mammary glands have been implicated in the development of breast cancer [34, 35]. Our data of over 18 patients from locally derived tissue, as well as 39 samples from CHTN, show that age has no significant effect on the distribution of the three mammary epithelial cell populations. While there are few human studies investigating the normal mammary gland in the context of aging, some have demonstrated a decline of myoepithelial cells and an increase in luminal cells expressing myoepithelial markers [8]. Our study also shows a modest increase in the LP population with aging, which appears consistent with the published studies. In mice, age is associated with an increase in myoepithelial and a reduction in possibly undifferentiated luminal cells among the epithelial populations [36] and marmosets were shown to have a reduction in luminal progenitors in aged individuals [37]. However, the reasons for the population shifts are still not understood and could depend on undifferentiated populations of adult cells that alter epithelial identity with age. Some mouse studies have shown a decline in facultative MaSCs, which might be credited to an accumulation of DNA damage, while others have implicated a decline in *Notch* signaling in age-related MaSC regression [38, 39]. Similarly, our data also show an age-dependent decline of human myoepithelial MaSC frequency, which is at least in part associated with menopause. This is understandable as hormonal signaling is known to increase MaSC frequency, particularly for progesterone, which is deficient after menopause and likely modulates stem cell activity through the RANK ligand paracrine signaling [40, 41]. How these stem and progenitor cells differ in aging or during menopause could reveal clues to prevention of age-related disorders and the mechanisms of age-associated changes are still undetermined. Clues are offered that point toward stem cells being more likely to undergo oncogene-induced malignant transformation due to their ability to control chromosomal instability, an event linked with aging [42]. Basal and luminal stem cells are also shown to possibly be the respective precursors to breast cancer stem cells that may contribute to mammary tumorigenesis [43]. The link between aging, MaSCs, and breast cancer makes a compelling case for further research in cancer prevention.

Our study shows that human MaSC activity is abrogated when mTOR signaling is inhibited. This observation appears intriguing as the myoepithelial population was not reduced by sirolimus treatment, yet the MaSCs derived from it showed the greatest abrogation, particularly in the postmenopausal patients. On the other hand, the frequency or activity of luminal progenitors as reflected by their sphere formation efficiency was not decreased even though the LP cell population was decreased by the treatment. However, through serial passaging of their organoids the luminal progenitor lifespan was significantly reduced by the treatment. While we do not have a mechanistic explanation as to why BM and LP cells show different responses to sirolimus treatment with respect to their population frequency and their SFE, some conjecture can be surmised. One is that some BM cells consist of bipotent stem cells who’s role is not primary for normal homeostatic turnover, but toward tissue development and regeneration/repair [44]. As such, BM cells are normally quiescent, and their number was not reduced by sirolimus treatment. On the other hand, the treatment might have driven them deeper into quiescence or even senescence such that many of them did not divide to form spheres. LP cells, in contrast, are the primary populations of hormonal and physiological turnover, which might have been inhibited by sirolimus due to cell cycle arrest, resulting in the reduced cell number. The inhibitory effect of sirolimus on LP cells appeared short lived such that when their SFE became normal after sirolimus treatment was stopped for 3–7 days before they were isolated for the SFE assay.

Our results contradict studies in mice and human cell lines in which mTOR inhibitors prevent a decline in adult stem cell function and increase their lifespan [45]. It should be stressed that our study incorporated treatment of human patients and did not rely on mouse strains or secondary cultured human cell lines, making the implications toward clinical research more germane. Further conflicts in other models include the use of mTOR inhibitors in mouse strains, which showed no ability in preventing mammosphere formation [46]. In contrast, primary tissue-derived human CD44^+^ EpCAM^+^ ALDH1^high^ ERα^−^ cancer stem cells showed an abrogation by mTOR inhibitors during combination treatment with tamoxifen, which failed to prevent sphere formation alone until an mTOR inhibitor was introduced alongside it [47]. This similarity to our own normal adult MaSCs’ sphere generation in response to the treatment suggests a common, targetable mechanism between the cancer and normal stem cells.

Our data showed that postmenopausal epithelial cells responded more significantly to sirolimus compared to pre-menopausal epithelial cells. This could be due to the stimulation of stem/progenitor cells by the hormonal signaling, particularly estrogen and progesterone, that is higher in pre-menopausal women. Postmenopausal cells without this hormonal stimulation may be more sensitive to the inhibition by mTOR signaling disruption. Among the pre-menopausal patients themselves, it would be interesting to investigate further if the menstrual cycles themselves could affect the expression of biomarkers in response to sirolimus treatment. Unfortunately, our pre-menopausal sample size was not sufficient to properly segregate and analyze such effect. As to the possible effects this might have, there is reason to believe that the cycle associated with high hormonal signaling, particularly progesterone during the luteal phase, to promote breast cell proliferation and growth would likely counteract the effects of mTOR inhibition on growth reduction [48]. Likewise, the effects of mTOR inhibition may reduce the epithelial response to hormonal signaling.

In relating our findings to a possible link in cancer, our data show that cancer progression markers are alleviated in tissue from treated patients. All patients were diagnosed with early-stage breast cancer whose prognosis is correlated with the cancer progression markers p16, COX2, and Ki67 [12]. Interestingly, the inhibition of the progression markers was not constrained to just DCIS, but also in normal ducts with the exception of Ki67 which was basically absent in normal ductal cells. The sirolimus treatment showed a distinct inhibitory effect on the mTORC1 kinase ability to phosphorylate p70S6K1 and 4E-BP1 in the mammary ducts, which is known to prevent nucleotide and protein synthesis [49]. Both are key components for the growth and proliferation of stem and progenitor cells for the generation of mammary epithelial tissue. Among the three markers, p16 is reported to have the strongest correlation with cancer progression. Its alleviation in both normal and early neoplastic tissue types provides a promising indication that sirolimus can be used in cancer prevention. While p16 is involved in cell cycle regulation, its overexpression is a notable factor in both senescence and cancer [50]. p16-positive cells likely promote a pro-tumorigenic environment through SASP-related factors including pro-inflammatory cytokines, chemokines, and growth factors [33]. Our study shows that common pro-inflammatory and SASP-associated factors, TNFα and IL-6, were both significantly reduced by sirolimus treatment in both normal and early cancer, which confirms a role of mTOR signaling in promoting the secretion of SASP factors. The pro-inflammatory environment also coincides with the presence of COX2, which is an enzyme that promotes inflammation within its resident tissue. This provides a scenario in which a pro-inflammatory environment is coupled with an increase in cell growth and proliferation resulting in neoplastic transformation of vulnerable cell types such as stem cells. Further investigation into the possible roles that mTOR could play in this scenario could lead to future preventative measures in preventing breast cancer development.

Although evidence for inflammation-associated genetic alterations and consequently carcinogenesis in cancer tissue is considerable [51], there is still no concordant indication that the adjacent normal tissue is likewise affected, particularly when cancer-associated genes are considered [52]. This suggests that susceptibility to mutagenic effects from tumor tissue might be countered by protective mechanisms that remain functional in nearby normal tissue. The fact that sirolimus treatment was effective in abrogating SASP-related gene expression and increase CDK inhibitor p21 in normal ducts indicates its utility in augmenting counter-neoplasia ability of adjacent normal tissues for secondary chemo-prevention. This notion is further strengthened by our finding that the sirolimus regimen did not appear to stimulate autophagy, which is known to be stimulated by mTOR inhibition [53]. The foci of the two autophagic markers p62 and LC3B were not significantly increased after the sirolimus treatment. LC3B foci were in fact modestly decreased in adjacent normal ducts of some cases. The absence of autophagy induction might be due to the short-term and low dose of sirolimus treatment plus the 3–7 day washout time. As autophagy has been reported as a possible survival mechanism of DCIS cells for progression to invasive tumors [54], the lack of or decreased autophagy after sirolimus treatment suggests that sirolimus may not induce this unwanted side effect for chemo-prevention when intermittently used for short term and at a low dose.

Another interesting finding was that nuclear staining of p21 was increased in normal epithelial cells after sirolimus treatment. It has been generally regarded that increased p21 expression in the nucleus is associated with its anti-tumorigenic function through cell cycle arrest [55]. The transformed cells in DCIS lesions showed no difference between treatments and might have acquired resistance to cell cycle inhibition by sirolimus, not present in the normal cells. The role of p21 in the progression of DCIS toward invasive carcinoma is not well understood although it is considered as a tumor inhibitor so long as other participants in the regulation of cell cycling, particularly p53, are functional [56]. In regard to stem cells, p21 is thought to promote stem cell longevity by restricting their proliferation and renewal in multiple systems [56]. If so, the inhibition of MaSCs by sirolimus may prevent malignant transformation as well as preserve their stemness capacity in breast tissue.

In conclusion, this study has shown an age-/menopause-dependent decline of MaSC activity and a possible use of sirolimus, a rapamycin analog, as a potential way to inhibit MaSC activity and self-renewal capacity of LP cells. While sirolimus has many side effects, particularly used at high doses for long term as an immunosuppressor, our study showed that the low-dose, short-term treatment appeared well tolerated with minor side effects and effective in countering SASP-associated inflammation and proliferation in early breast tumor microenvironment. Thus, our study provides a proof of principal for potential development of MTOR inhibitors as primary and/or secondary chemo-preventive agents. More studies are clearly needed to determine whether intermittent use of rapamycin analogs at low doses for long term is effective in preventing breast cancer in high-risk populations with acceptable side effects.

### Supplementary Information

**Additional file 1**: **Figure S1:** Representative FACS analyses of EpCAM and CD49f expression in cells isolated from control and treated patient samples. Gates identifying luminal progenitor (LP), mature luminal (ML), and basal myoepithelial (BM) populations are shown which were derived from Lin^−^ gating using Streptavidin-Brilliant Violet 605. FITC, fluorescein isothiocyanate; APC, Allophycocyanin.

**Additional file 2**: **Figure S2: **Frequency of epithelial cell populations in the mammary gland from women with varied age. Linear regression analysis showing changes in proportions of BM cells (A & B), LP cells (D & E), and ML cells (G & H) as a function of age for CHTN (n= 39 individuals) and local (UT Control) (n=18) patient samples. Panels C, F, and I show the distribution of the proportions of these three cell types in pre- (n=8) and postmenopausal (n=10) local patient samples with P values from unpaired t-tests. Each dot represents one patient.

**Additional file 3**: **Figure S3:** SFE of BM and LP cells from women with varied age. Linear regression showing the SFE of BM cells in CHTN and local samples (A & B) from FACS sorting of epithelial populations as a function of age for CHTN (n= 39 individuals) and local samples (n=18). SFE values of pre- vs postmenopausal derived tissue are also shown (n= 8, 10) and differences calculated with unpaired t-test (C). LP SFEs are shown as a function of age (D & E) for both data sets (n=39,18) and as a function of menopausal status (F).

**Additional file 4**: **Figure S4:** Additional markers of mTORC1 activity and autophagy. (A) IHC images and quantification of breast tissue from normal (n=12) and DCIS ducts (n=8) of control and sirolimus treated patients for phospho-4E-BP1 (Thr37/46). Significance was evaluated by 2-way ANOVA. Scale bar, 100 μm. (B) IHC images and quantification for p62 staining from normal (n=12) and DCIS ducts (n=4). (C) IHC images and quantification of LC3B staining from normal (n=11) and DCIS ducts (n=6). Significance was evaluated by paired t-test. Scale bar, 20 μm. Instead,

**Additional file 5**: **Figure S5: **Quantification of p16 nuclear staining from IHC of pre- and post-sirolimus treated breast tissues. Quantification of breast tissue from normal (n=12) and DCIS ducts (n=12) of control and sirolimus treated patients for p16 nuclear staining. Significance was evaluated by 2-way ANOVA.

**Additional file 6**: **Table S1. **Summary of patient and tissue information

**Additional file 7**: **Table S2. **Multivariate analysis of variance

## Data Availability

The datasets used and/or analyzed during the current study are available from the corresponding author on reasonable request.

## Abbreviations

4E-BP1: EIF4E Binding Protein 1
ADH: Atypical Ductal Hyperplasia
AE: Adverse effects
ALH: Atypical Lobular Hyperplasia
BC: Breast Cancer
BV: Brilliant violet
CD44: Cluster of Differentiation 44
CHTN: Cooperative Human Tissue Network
DCIS: Ductal Carcinoma In Situ
EpCAM: Epithelial cell adhesion molecule
ER: Estrogen Receptor
FACS: Fluorescence-Activated Cell Sorting
FITC: Fluorescein isothiocyanate
IHC: Immunohistochemistry
LCIS: Lobular Carcinoma In Situ
LP: Luminal Progenitors
MaSCs: Mammary Stem Cells
mTORC1/2: Mammalian Target Of Rapamycin Complex 1/2
MRU: Mammary Repopulating Unit
p21: P21Cip1
p70S6K1: P70S6 Kinase 1
PBST: Phosphate-Buffered Saline Triton
PE: Phycoerythrin (PE)
PI3K: Phosphoinositide 3-Kinases
PR: Progesterone Receptor
RANK: Receptor Activator of NF-κB
SFE: Sphere formation efficiency
SASP: Senescence-Associated Secretory Phenotype
TNF: Tumor necrosis factor
