# Supplementary material for: mTOR inhibition abrogates human mammary stem cells and early breast cancer progression markers
Source: Breast Cancer Res. 2023 Oct 30;25:131. doi: 10.1186/s13058-023-01727-z (PMC10614399; doi:10.1186/s13058-023-01727-z)
Supplement: Supplementary file 1 — Additional file 1: Figure S1: Representative FACS analyses of EpCAM and CD49f expression in cells isolated from control and treated patient samples. Gates identifying luminal progenitor (LP), mature luminal (ML), and basal myoepithelial (BM) populations are shown which were derived from Lin− gating using Streptavidin-Brilliant Violet 605. FITC, fluorescein isothiocyanate; APC, Allophycocyanin. [file 13058_2023_1727_MOESM1_ESM.pptx]

## Slide 1
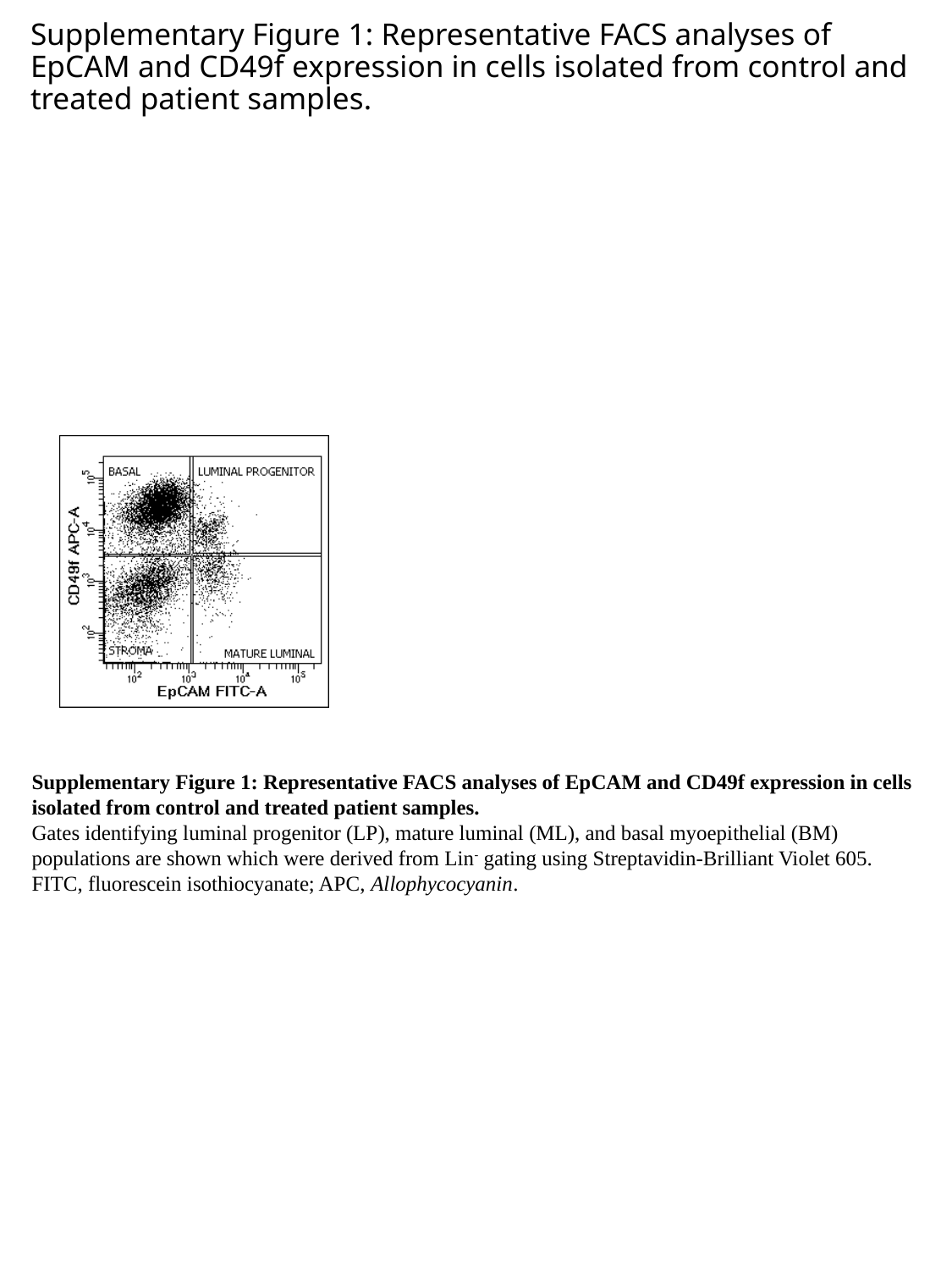

# Supplementary Figure 1: Representative FACS analyses of EpCAM and CD49f expression in cells isolated from control and treated patient samples.
Supplementary Figure 1: Representative FACS analyses of EpCAM and CD49f expression in cells isolated from control and treated patient samples.
Gates identifying luminal progenitor (LP), mature luminal (ML), and basal myoepithelial (BM) populations are shown which were derived from Lin- gating using Streptavidin-Brilliant Violet 605. FITC, fluorescein isothiocyanate; APC, Allophycocyanin.
