# Supplementary material for: mTOR inhibition abrogates human mammary stem cells and early breast cancer progression markers
Source: Breast Cancer Res. 2023 Oct 30;25:131. doi: 10.1186/s13058-023-01727-z (PMC10614399; doi:10.1186/s13058-023-01727-z)

## Slide 1
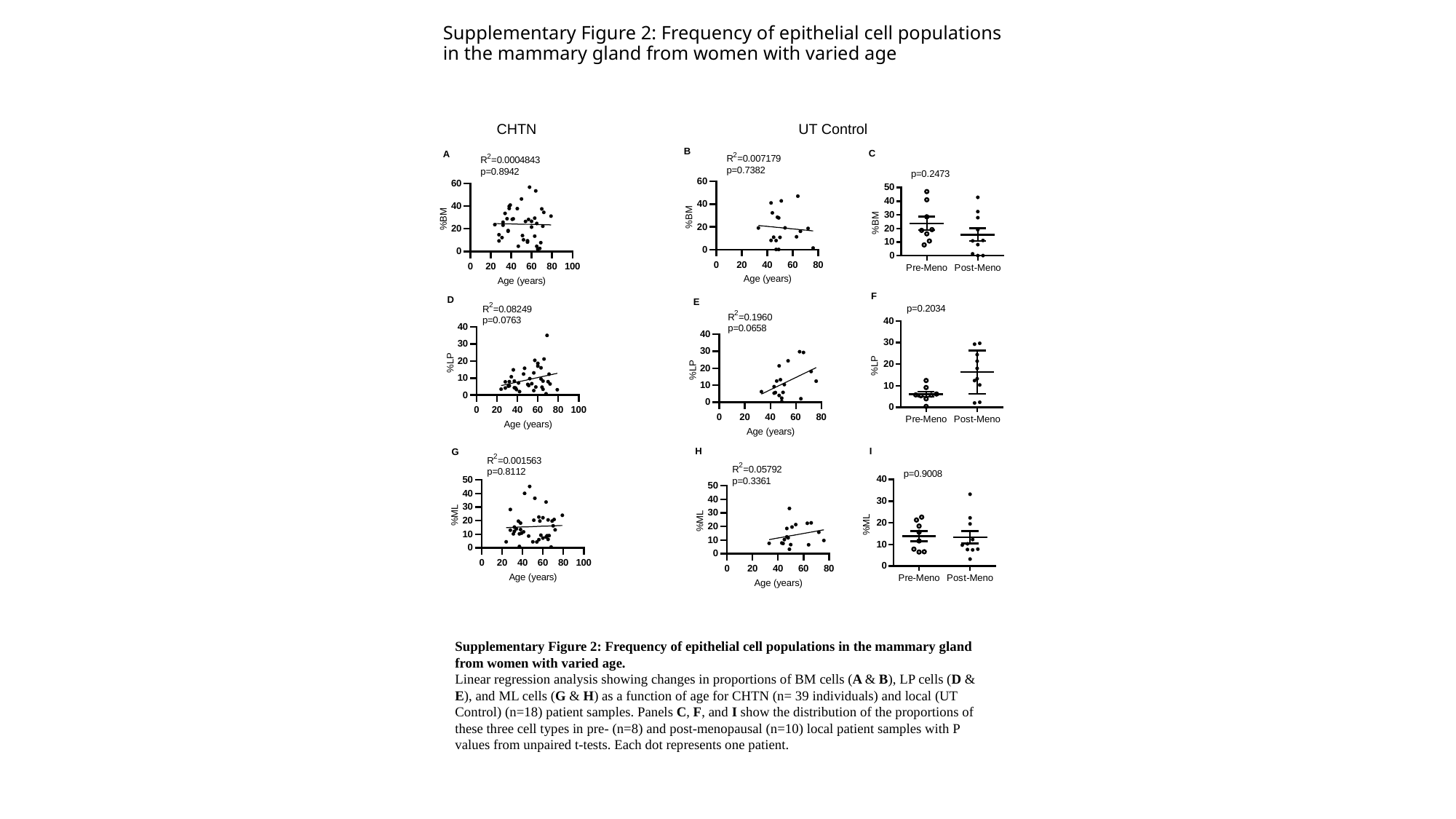

# Supplementary Figure 2: Frequency of epithelial cell populations in the mammary gland from women with varied age
CHTN
UT Control
B
C
A
F
D
E
H
I
G
Supplementary Figure 2: Frequency of epithelial cell populations in the mammary gland from women with varied age.
Linear regression analysis showing changes in proportions of BM cells (A & B), LP cells (D & E), and ML cells (G & H) as a function of age for CHTN (n= 39 individuals) and local (UT Control) (n=18) patient samples. Panels C, F, and I show the distribution of the proportions of these three cell types in pre- (n=8) and post-menopausal (n=10) local patient samples with P values from unpaired t-tests. Each dot represents one patient.

## Slide 2
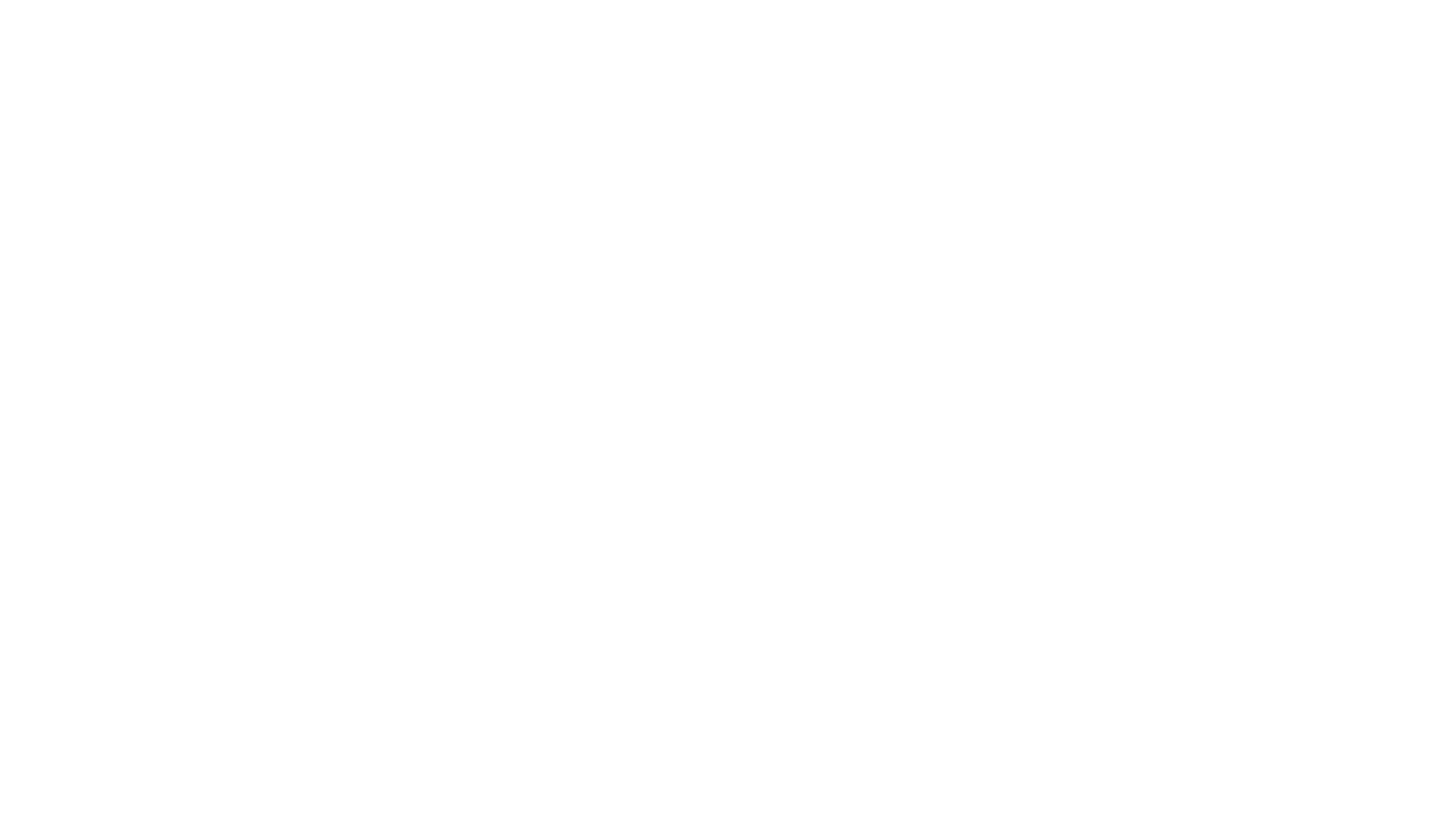

## Slide 3
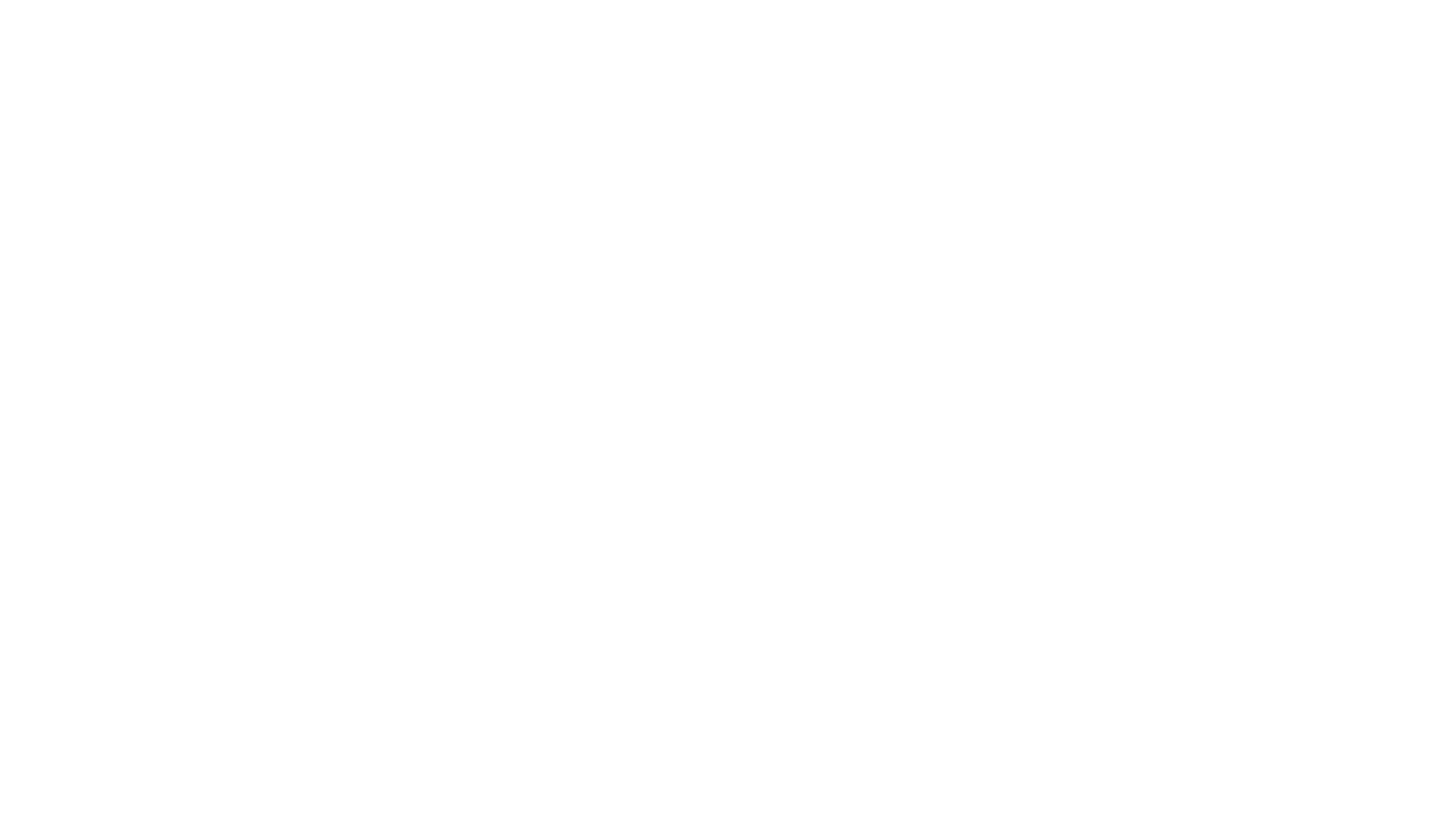

Supplement: Supplementary file 2 — Additional file 2: Figure S2: Frequency of epithelial cell populations in the mammary gland from women with varied age. Linear regression analysis showing changes in proportions of BM cells (A & B), LP cells (D & E), and ML cells (G & H) as a function of age for CHTN (n= 39 individuals) and local (UT Control) (n=18) patient samples. Panels C, F, and I show the distribution of the proportions of these three cell types in pre- (n=8) and postmenopausal (n=10) local patient samples with P values from unpaired t-tests. Each dot represents one patient. [file 13058_2023_1727_MOESM2_ESM.pptx]
