# Supplementary material for: mTOR inhibition abrogates human mammary stem cells and early breast cancer progression markers
Source: Breast Cancer Res. 2023 Oct 30;25:131. doi: 10.1186/s13058-023-01727-z (PMC10614399; doi:10.1186/s13058-023-01727-z)
Supplement: Supplementary file 3 — Additional file 3: Figure S3: SFE of BM and LP cells from women with varied age. Linear regression showing the SFE of BM cells in CHTN and local samples (A & B) from FACS sorting of epithelial populations as a function of age for CHTN (n= 39 individuals) and local samples (n=18). SFE values of pre- vs postmenopausal derived tissue are also shown (n= 8, 10) and differences calculated with unpaired t-test (C). LP SFEs are shown as a function of age (D & E) for both data sets (n=39,18) and as a function of menopausal status (F). [file 13058_2023_1727_MOESM3_ESM.pptx]

## Slide 1
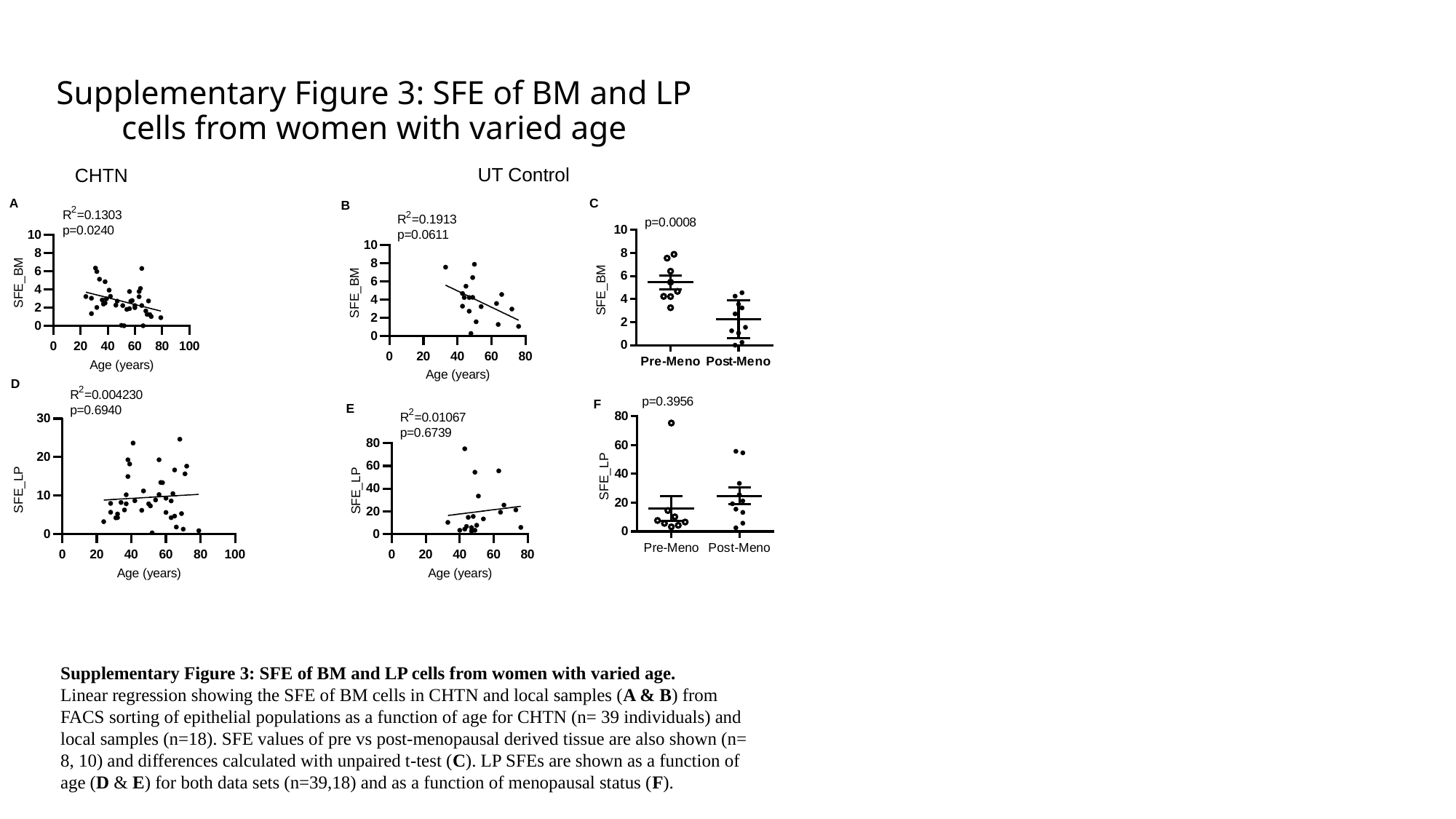

Supplementary Figure 3: SFE of BM and LP cells from women with varied age
UT Control
CHTN
A
C
B
D
F
E
Supplementary Figure 3: SFE of BM and LP cells from women with varied age.
Linear regression showing the SFE of BM cells in CHTN and local samples (A & B) from FACS sorting of epithelial populations as a function of age for CHTN (n= 39 individuals) and local samples (n=18). SFE values of pre vs post-menopausal derived tissue are also shown (n= 8, 10) and differences calculated with unpaired t-test (C). LP SFEs are shown as a function of age (D & E) for both data sets (n=39,18) and as a function of menopausal status (F).
