# Supplementary material for: mTOR inhibition abrogates human mammary stem cells and early breast cancer progression markers
Source: Breast Cancer Res. 2023 Oct 30;25:131. doi: 10.1186/s13058-023-01727-z (PMC10614399; doi:10.1186/s13058-023-01727-z)
Supplement: Supplementary file 4 — Additional file 4: Figure S4: Additional markers of mTORC1 activity and autophagy. (A) IHC images and quantification of breast tissue from normal (n=12) and DCIS ducts (n=8) of control and sirolimus treated patients for phospho-4E-BP1 (Thr37/46). Significance was evaluated by 2-way ANOVA. Scale bar, 100 μm. (B) IHC images and quantification for p62 staining from normal (n=12) and DCIS ducts (n=4). (C) IHC images and quantification of LC3B staining from normal (n=11) and DCIS ducts (n=6). Significance was evaluated by paired t-test. Scale bar, 20 μm. Instead, [file 13058_2023_1727_MOESM4_ESM.pptx]

## Slide 1
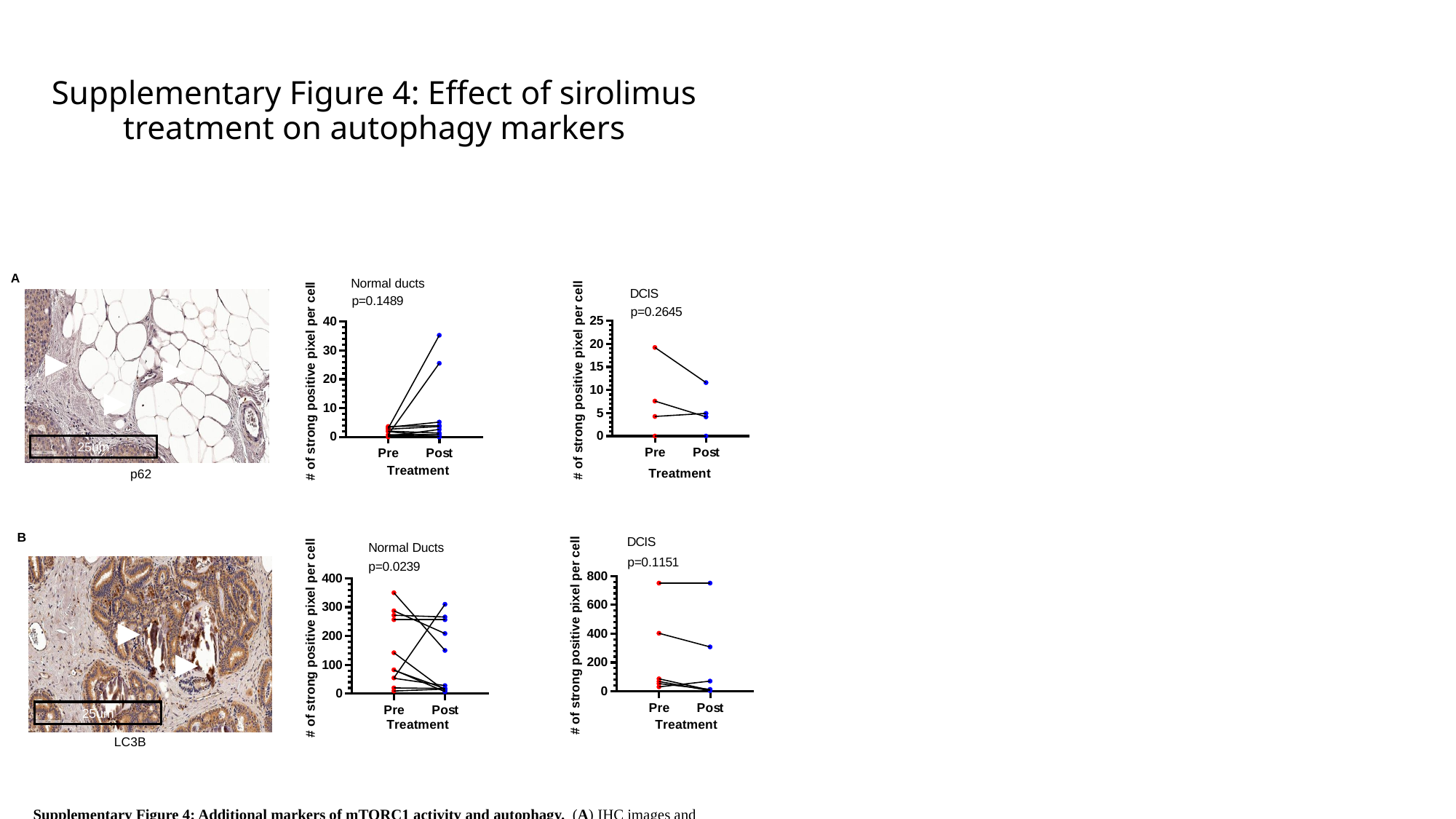

Supplementary Figure 4: Effect of sirolimus treatment on autophagy markers
A
25um
p62
B
25um
LC3B
Supplementary Figure 4: Additional markers of mTORC1 activity and autophagy. (A) IHC images and quantification for p62 staining from normal (n=12) and DCIS ducts (n=4). (B) IHC images and quantification of LC3B staining from normal (n=11) and DCIS ducts (n=6). Significance was evaluated by paired t-test. Scale bar, 20 µm.
