# Supplementary material for: mTOR inhibition abrogates human mammary stem cells and early breast cancer progression markers
Source: Breast Cancer Res. 2023 Oct 30;25:131. doi: 10.1186/s13058-023-01727-z (PMC10614399; doi:10.1186/s13058-023-01727-z)
Supplement: Supplementary file 5 — Additional file 5: Figure S5: Quantification of p16 nuclear staining from IHC of pre- and post-sirolimus treated breast tissues. Quantification of breast tissue from normal (n=12) and DCIS ducts (n=12) of control and sirolimus treated patients for p16 nuclear staining. Significance was evaluated by 2-way ANOVA. [file 13058_2023_1727_MOESM5_ESM.pptx]

## Slide 1
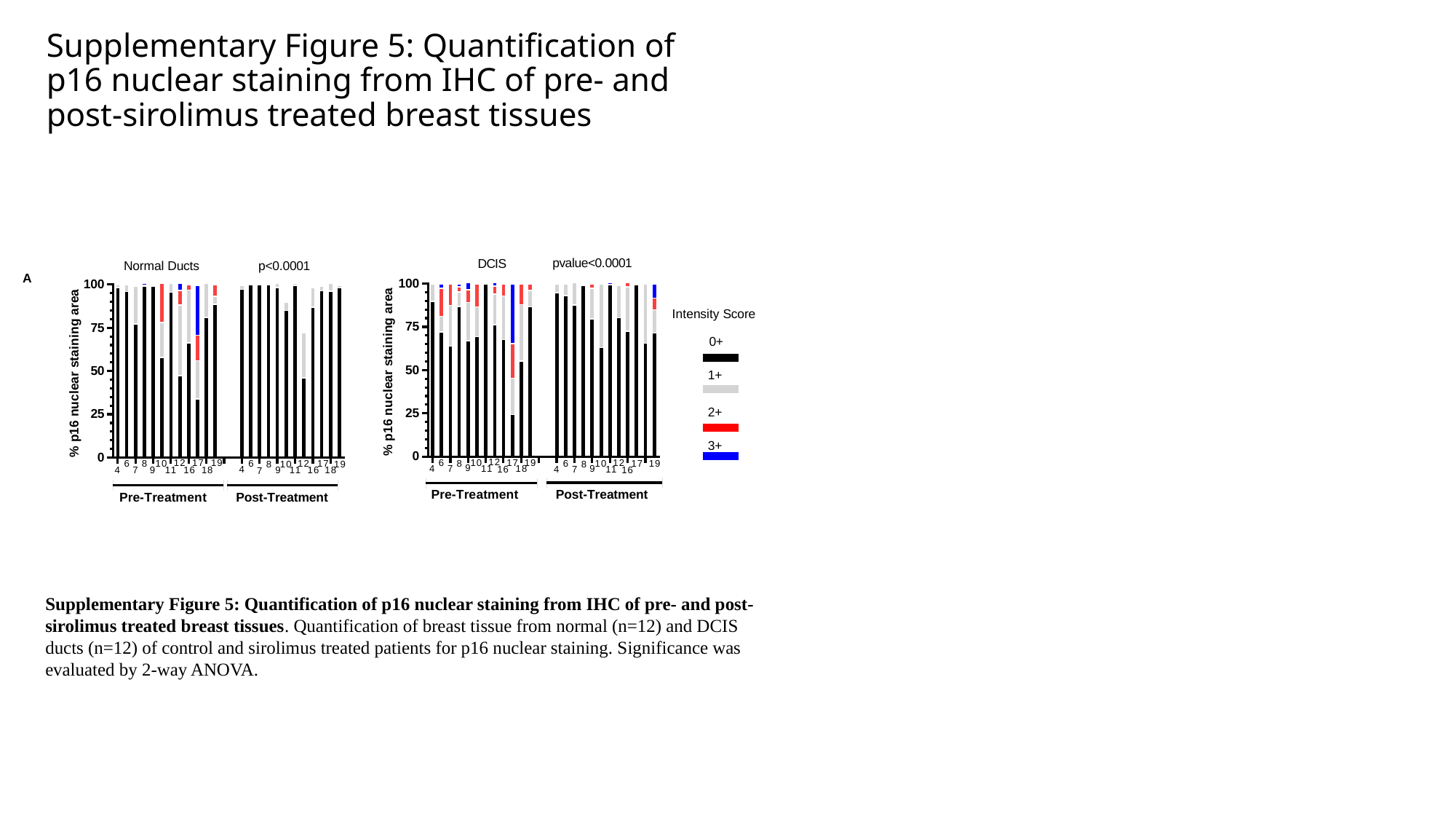

Supplementary Figure 2: Additional markers of mTORC1 activity and autophagy
Supplementary Figure 5: Quantification of p16 nuclear staining from IHC of pre- and post-sirolimus treated breast tissues
A
Supplementary Figure 5: Quantification of p16 nuclear staining from IHC of pre- and post-sirolimus treated breast tissues. Quantification of breast tissue from normal (n=12) and DCIS ducts (n=12) of control and sirolimus treated patients for p16 nuclear staining. Significance was evaluated by 2-way ANOVA.
